# Working memory and processing speed training in schizophrenia: study protocol for a randomized controlled trial

**DOI:** 10.1186/s13063-016-1188-5

**Published:** 2016-01-26

**Authors:** Briana D. Cassetta, Vina M. Goghari

**Affiliations:** Clinical Neuroscience of Schizophrenia Laboratory, Administration Building, Department of Psychology, University of Calgary, 2500 University Drive NW, Calgary, AB T2N 1N4 Canada

**Keywords:** Schizophrenia, cognitive remediation, working memory, processing speed, randomized controlled trial, study protocol

## Abstract

**Background:**

In most domains of cognition, individuals with schizophrenia are generally found to be one standard deviation below the mean of the controls. As a result, examining the impact of cognitive remediation in individuals with schizophrenia has been a burgeoning area of research. However, the state of the literature remains unclear as to which domains of cognition should be targeted to produce the most widespread and durable benefits for individuals with schizophrenia. One suggestion is that targeting lower-level cognitive processes that are important for higher-level and more complex aspects of cognition may produce the most widespread benefits in cognition and everyday functioning. Relatively few studies have examined the effects of working memory or processing speed training in schizophrenia, as most studies examine broad-based remediation programs. Thus, a need exists for targeted working memory and processing speed training studies to better understand the mechanisms of cognitive enhancement in patients. This study aims to 1) investigate near-transfer gains (that is, the transfer of learning to related contexts) associated with working memory and processing speed training in schizophrenia patients; 2) investigate far-transfer gains (that is, the transfer of learning to new contexts) associated with working memory and processing speed training (that is, gains in other neurocognitive domains and social cognition); and 3) investigate real-world gains associated with training (that is, gains in daily functioning).

**Methods/Design:**

A double-blind randomized controlled trial with a three parallel group design will be conducted. A random sample of 81 patients with schizophrenia or schizoaffective disorder will be recruited through outpatient clinics at Foothills Hospital and community support programs in Calgary, Alberta. Participants will be randomly assigned using a computer-generated program in a 1:1:1 ratio to a working memory-training group, a processing speed-training group, or a no-training control group. Training will be completed at home for 30 minutes per day, 5 days per week, for a total of 10 weeks. Neurocognitive, social cognitive, and daily functioning measures will be administered both pre- and post-training to detect training-related gains. The primary outcome measures will include working memory and processing speed (near-transfer measures), as well as fluid intelligence (far-transfer measure).

**Trial registration:**

Current controlled trials NCT02478827 (ClinicalTrials.gov, registered on 15 June 2015).

**Electronic supplementary material:**

The online version of this article (doi:10.1186/s13063-016-1188-5) contains supplementary material, which is available to authorized users.

## Background

### Introduction

Schizophrenia is characterized by a variety of symptoms that impact the normal experience, such as hallucinations, delusions, disorganized speech, disorganized behavior, apathy, and emotional flatness [1]. In addition to these defining features, studies on individuals with schizophrenia consistently show that cognitive functioning is poor and remains poor throughout the course of the illness [2]. These impairments relate to day-to-day functioning, such as vocational and social functioning, in patients [2]. Given the significant impact of cognitive deficits on daily living in individuals with schizophrenia, researchers have begun to explore methods to remediate cognitive deficits. Specifically, cognitive remediation therapy is designed to improve neurocognitive abilities through drill and practice and the introduction of compensatory and/or adaptive strategies, with the ultimate aim of improving cognitive abilities such as attention, memory, and problem solving [3]. Several studies have examined the effects of cognitive remediation therapy on many aspects of neurocognition in schizophrenia patients [4, 5]. However, the majority of these studies have examined the effects of broad-based cognitive interventions, leaving many unanswered questions about the active components of training. In other words, which domains of cognition should be trained to produce the most widespread benefits in cognition and functioning in individuals with schizophrenia? As such, there is a need for more research examining both the near-transfer (that is, the transfer of learning to related contexts) and far-transfer (that is, the transfer of learning to new contexts) gains associated with cognitive remediation of specific domains of cognition in schizophrenia patients.

For the purposes of the current study, working memory and processing speed deficits in schizophrenia will be examined more closely. Importantly, recent literature has underscored the need for further examination of working memory and processing speed training specifically in schizophrenia patients, given their important roles in higher-order cognitive performance as well as everyday functioning [6]. Thus, the goal of the current study is to examine both the near-transfer and far-transfer effects of working memory and processing speed training in schizophrenia patients.

### Cognitive impairments in schizophrenia patients

Schizophrenia is associated with a wide range of symptoms impacting a number of different domains. In addition to the symptoms of schizophrenia used in diagnosis, the disorder is generally accompanied by a broad array of impairments in cognition. The extent of these impairments is generally quite significant, with many studies finding cognitive abilities in schizophrenia patients to be 1 to 2 standard deviations lower than healthy controls across multiple domains [7, 8]. The profile of cognitive impairments includes many significant aspects of human cognition, including memory, processing speed, attention, executive functioning, and social cognition [9]. In addition, a general deficit in intelligence is commonly found in individuals with schizophrenia [10].

Whether the neurocognitive deficits in schizophrenia are specific to domains or generalized across domains is a debated issue in the cognitive literature. However, recent research has shown that, in addition to the specific cognitive impairments found in schizophrenia patients as described above, a more general deficit exists in global intelligence [10]. Importantly, deficits in this general factor (that is, intelligence) account for a significant portion, but not all, of the cognitive impairment in this patient group [10]. Rather, cognitive impairments in specific domains (for example, working memory and processing speed) are likely superimposed on a more general cognitive deficit in schizophrenia. In fact, schizophrenia patients are shown to have worse neurocognitive functioning than healthy controls with the same level of general cognitive functioning (that is, IQ) [11].

Importantly, cognition has been linked to daily functioning and real-world skills in schizophrenia patients. In fact, all of the main neurocognitive constructs have been linked to some aspect of functional outcome in schizophrenia, typically with medium-sized effect [2, 12]. More specifically, cognitive impairments in schizophrenia have been related to worse performance at work and greater unemployment [13], decreased ability to live independently [14], reduced subjective rating of quality of life [15], higher rates of medication mismanagement and noncompliance [16], and increased medical comorbidity [17]. Notably, working memory, processing speed, and fluid intelligence have all been linked to multiple aspects of daily functioning in schizophrenia patients.

### Cognitive remediation in schizophrenia

Given the array of cognitive deficits associated with schizophrenia and their relation to daily functioning, numerous research groups have examined the impact of cognitive remediation on many aspects of cognition (for example, attention, memory, executive functions, social cognition, and fluid intelligence). At the 2010 Cognitive Remediation Experts Workshop, cognitive remediation was described as a behavioral-training intervention with the aim of improving cognitive abilities and a specific emphasis on both generalizability and durability [18]. Several reviews and meta-analyses have been published on the benefits of cognitive remediation in schizophrenia patients [4, 5]. Results from these meta-analyses typically indicate promising results from cognitive remediation programs. For example, one meta-analysis examining 26 studies (1,151 subjects) found significant effect sizes for overall cognition, as well as most individual domains of cognitive functioning, including effect sizes of *d* = .41 in global cognition, *d* = .41 in attention, *d* = .48 in processing speed, *d* = .52 in verbal working memory, and *d* = .47 in reasoning/problem solving [4]. Similar effect sizes were found in a more recent meta-analysis examining the effects of cognitive remediation (for example, effect size of *d* = .45 in global cognition) [5].

However, which domain(s) of cognition should be targeted to produce the most widespread and durable benefits for schizophrenia patients is not clear. It may be that targeting lower-level cognitive processes that are important for higher-level and more complex aspects of cognition may produce the most widespread benefits in cognition and everyday functioning for patients. Given that neuropsychiatric illnesses such as schizophrenia are associated with abnormal learning mechanisms in the brain, researchers have suggested that cognitive training should focus on *specific* changes in neural representations and processing efficiency [19]. More specifically, it has been postulated that training low-level (for example, pre-attentive perceptual processing) and mid-level (for example, working memory) cognitive process are necessary for improving high-level cognitive processes (for example, recognizing facial emotions) [19]. Logically, targeting specific lower level cognitive processes may also be a more efficient means of obtaining treatment gains. Targeting cognitive processes with broad associations to other aspects of cognition and functioning may be the best strategy for treatment with schizophrenia patients [20]. This approach would plausibly lead to enhancements in multiple domains of cognition by efficiently targeting one broad-reaching domain. Consistent with this, research has shown that improvements in lower level processing through learning can induce large-scale changes in cortical representations associated with higher-order cognition [19].

Given that working memory has been associated with the likes of cognitive control, planning, fluid intelligence, and daily functioning in schizophrenia patients, one might logically predict that a training program specifically targeting working memory processes would lead to more generalized improvements in the aforementioned domains of cognition and functioning. Similarly, as described above, processing speed has been shown to be an important mediator in most aspects of cognition and, thus, it is reasonable to hypothesize that an intervention targeting processing speed specifically might lead to more widespread gains in multiple cognitive abilities and areas of functioning.

### Working memory

Working memory describes the ability to actively hold multiple pieces of information in mind and mentally manipulate this information over short periods. Working memory has been strongly related to executive functioning (for example, inhibition, mental flexibility, and planning) [21]. Various models of working memory have been postulated in the literature [22–24]; however, most models describe working memory as the temporary storage and processing of new information in both the verbal and visual domains.

#### Working memory in schizophrenia

Despite the diverse methods and theoretical approaches to examining working memory abilities across the schizophrenia literature, meta-analyses suggest that working memory deficits are reliably found across these varying approaches [25, 26]. In fact, working memory has been described as a core cognitive deficit associated with schizophrenia [27] and not simply attributable to IQ deficits [25]. In addition, working memory deficits have been found in unaffected first-degree biological relatives of schizophrenia patients [28] and in healthy individuals with characteristics of schizophrenia, such as high levels of social anhedonia or magical ideation/perceptual aberration [29]. This suggests that working memory impairments are a strong candidate for being a biological marker of schizophrenia.

Furthermore, the working memory deficit is found in schizophrenia patients regardless of the task modality (that is, visuospatial or verbal) or the duration of the delay periods [26]. Working memory abilities have been related to numerous other cognitive domains that are impaired in schizophrenia, such as memory, attention, and planning [27], and other executive functions such as inhibitory control and mental flexibility [21], as well as functional outcomes, such as job tenure and symptom severity [27]. In addition, working memory is related to fluid intelligence. Fluid intelligence is the ability to think logically and solve problems in novel situations through reasoning and is distinguished from acquired knowledge, or crystallized intelligence [30]. Fluid intelligence is critical for a wide range of cognitive tasks and is closely related to professional and educational success [31]. Measures of fluid intelligence have been shown to be highly correlated with working memory abilities (that is, correlations ranging between *r* = 0.20 and *r* = 0.90 across studies) [32]. Though working memory abilities and fluid intelligence are highly correlated, most researchers concur that they are dissociable constructs, with fluid intelligence being a higher-order cognitive domain requiring inferential reasoning to understand associations and solve problems [32, 33]. However, both working memory and fluid intelligence share common capacity limits and neural correlates, suggesting that they are cognitively related [32].

Moreover, working memory has been related to aspects of social cognition (for example, processing of social situations) [34]. In fact, memory training has been shown to improve social perception in schizophrenia patients [35], which provides preliminary evidence for the benefits of working memory training on social cognition. Thus, remediation of working memory deficits should be a goal in the treatment of schizophrenia patients. As discussed above, many different models of working memory have been described in the literature [23, 24, 36]. An examination of ten different working memory models by Miyake and Shah [37] identified a commonality among most models: the temporary storage and processing of information. As such, working memory training attempts to improve an individual’s ability to temporarily store and process information.

### Working memory training

#### Healthy individuals

Some research with healthy persons has shown that working memory training can indeed be effective at improving cognitive performance. More specifically, several studies have shown that working memory training results in near-transfer gains (that is, where transfer contexts and utilized skills are similar), such as improvements in recognition memory, immediate recall, and other working memory tasks [38, 39]. Near-transfer gains have been shown to remain stable 18 months after training in healthy young adults [40].

Moreover, researchers have examined far-transfer gains (that is, where transfer contexts and utilized skills are dissimilar) of working memory in healthy persons. Fluid intelligence is the most commonly studied far-transfer outcome measure in examinations of working memory training [20, 33]. As described above, fluid intelligence and working memory have been shown to be highly related constructs. In concordance with these findings, several studies have found far-transfer gains in fluid intelligence following working memory training in healthy individuals [31, 33, 41]. These far-transfer gains have been proposed possibly to be due to the adaptive nature of the training, thereby leading to the continual engagement of executive processes, the involvement of attentional control (which has been shown to be essential for both working memory and fluid intelligence), or simply the increase in working memory capacity, which may be crucial in fluid intelligence tasks [31].

In addition to gains in fluid intelligence, results of working memory-training studies have found far-transfer gains in other domains of cognition, including cognitive control (measured with the Stroop task) and reading comprehension [42], suggesting that working memory training can have an impact on a more domain-general mechanism (for example, executive attentional processes, gate control of information, interference control mechanisms, and/or engagement of specific domain strategies) [43].

However, other studies have not found evidence of near- or far-transfer gains following working memory training with healthy individuals [44–46]. Given the mixed findings in the working memory-training literature, a great deal of controversy exists around whether working memory training can have transfer effects to improve cognition, particularly far-transfer. For example, there have been mixed findings on whether working memory training can improve intellectual abilities (that is, fluid intelligence). In a recent review [47], six studies were found to report improvements in reasoning or learning following working memory training [31, 48–52], while five studies found no change in reasoning abilities [40, 42, 53–55].

Inconsistencies across studies might be due to the variety of comparison groups used (for example, active control, wait-list control), variability in training programs, and variability in transfer skills that are assessed. Notably, the positive results of working memory-training studies are controversial and have been criticized by several research groups. Criticisms include lack of random assignment in some studies, lack of suitable control groups, and relying on individual tasks to measure an entire construct [47, 56]. Thus, the interpretation of positive findings in the aforementioned studies remains disputed, with critics calling for more research with both active and no-contact control groups, as well as multiple measures for each construct of interest. Additionally, differences in baseline cognitive performance may also account for variability in study findings. Importantly, it has been suggested that high baseline intelligence may prevent the occurrence of training related gains due to ceiling effects [20]. As such, one might expect the benefits of cognitive training, including far-transfer gains, to be different in an individual with neuropsychiatric illness from in a healthy individual.

#### Individuals with schizophrenia

Relatively few studies have examined the effects of working memory training on individuals with schizophrenia; however, studies that have been completed do show promising results. For example, one research group reported improvements in visual working memory, verbal working memory, and visual short-term memory in chronic schizophrenia patients following just 4 weeks of computerized working memory training (that is, verbal and visuospatial working memory tasks), compared to a control group without intervention [57]. In another study, a computerized training program aimed at improving lower-level auditory processing (for example, distinguishing between sound frequencies, phonemes, and syllables) in addition to auditory-verbal working memory and verbal learning (for example, remembering verbal instructions and details from conversations) was compared to a control computer game program with schizophrenia patients [58]. The results of this study showed that individuals with schizophrenia receiving the active training program, as compared to the control program, had significant gains in verbal working memory, verbal learning and memory, as well as global cognition after 10 weeks of training [58]. Notably, the effect sizes found in this auditory working memory-training study were greater than the majority of studies reported in a recent meta-analysis by McGurk and colleagues [4] on cognitive remediation in schizophrenia patients. Given that the studies reported in the meta-analysis were typically broad-based examinations of cognitive remediation involving multiple domains, it may be the case that targeted working memory training is more effective at improving both working memory abilities, as well as general cognition [4, 58].

A handful of studies have also examined the neural changes associated with behavioral improvements on working memory tasks in schizophrenia patients following cognitive training. These studies provide further support for the benefits of cognitive remediation on working memory performance. One research group examined the effects of targeted verbal working memory training using a serial position verbal memory task on working memory and memory performance [59]. Following 10 weeks of training, three out of eight patients showed significant improvements in verbal working memory, and gains were associated with increased activation of the left inferior frontal cortex − the same region that is activated during verbal memory tasks in healthy individuals [59]. Additionally, other research groups have examined more broad cognitive training interventions on impacting working memory abilities. In one study, cognitive training in attention, working memory, logical thinking, and executive functioning domains led to greater activation in the prefrontal cortex on a spatial working memory task compared to a group that did not receive cognitive training [60]. Similarly, another research group showed increased activation in regions of the prefrontal cortex (that is, the dorsolateral prefrontal cortex, anterior cingulated, and frontopolar cortex) along with associated improvements in attention and working memory following a cognitive remediation program targeting attention and working memory in schizophrenia patients [61].

Thus, current research shows promising results for restoring working memory abilities following cognitive remediation in schizophrenia patients; however, given that the training programs examined are typically broad-based, it remains difficult to determine which aspects of training are providing the benefit. Given that working memory abilities are vitally important for day-to-day functioning and social interactions, there is a need for more targeted training programs with larger sample sizes to disentangle the role of each training component on improving working memory and other aspects of cognition. Moreover, it has been suggested that utilizing working memory training with a population known to have significant cognitive deficits might lead to greater benefits than those seen in healthy populations with little or no cognitive difficulties [20].

### Processing speed

Processing speed refers to the number of correct responses that an individual is able to make in a task during a specified amount of time. Thus, it is the ability to process information rapidly, and has been associated with broadly reduced volumes of gray matter in the prefrontal and temporal regions, as well as broad white matter alterations [62]. Many higher-order operations, such as perceptual processing, encoding and retrieval processes, and decision-making operations, are dependent on processing speed to some extent [62]. Similarly, processing speed and fluid intelligence are significantly correlated with one another (that is, correlations typically ranging from *r* = 0.30 to *r* = 0.40) [63]. More complex models indicate that processing speed, working memory, and fluid intelligence are all inter-related, with speed being a determinant of working memory capacity, and both speed and working memory being determinants of fluid intelligence [30]. The general significance of processing speed is evident by its inclusion in many measures of general intelligence [64].

#### Processing speed in schizophrenia

Recent meta-analyses have highlighted processing speed as being the central cognitive deficit in schizophrenia, with greater effect sizes than any other neurocognitive domain [62, 65]. Moreover, processing speed has been related to the deficit in emotion recognition in individuals with schizophrenia [66]. Specifically, processing speed is consistently shown to be a disproportionate deficit in schizophrenia against a backdrop of a generalized cognitive deficit [62, 67]. As such, it has been hypothesized that processing speed might mediate a broader array of cognitive impairments in schizophrenia [68]. Specifically, processing speed deficits have been shown to mediate impairments in attention, executive functions [68], verbal memory, verbal fluency, social cognition and functional outcomes [69]. While studies show that processing speed deficits can be partially explained by medication (for example, chlorpromazine) dosage, the evidence is also clear that processing speed deficits remain substantial even after accounting for medication effects [70]. In addition, impaired processing speed has been found in first-degree relatives of schizophrenia patients, as well as in individuals at high risk of developing the illness [62], suggesting that processing speed might be another biological marker of schizophrenia.

Importantly, processing speed correlates with numerous important clinical features in schizophrenia, including job tenure [71], self-care management, social functioning [72], and independent living [62]. Logically, increased response latency in social interactions likely hinders social relationships and social information processing. In addition, processing speed has been described as likely being the best longitudinal predictor of level of autonomy in patients with chronic schizophrenia [72]. Taken together, processing speed has a graded relationship with risk for schizophrenia, presence of schizophrenia, severity, and functional outcome, which supports the need for remediation efforts to address processing speed impairments.

### Processing speed training

#### Healthy individuals

Processing speed training has the primary aim of improving mental processing of increasingly complex information that can be processed accurately within briefer periods. The majority of the processing speed training literature in healthy populations focuses on older adults. For example, in a paper analyzing six different studies using a visual information processing speed-training paradigm, the authors found that speed of processing improved among 55 to 95 year olds, and that benefits of training lasted for at least 2 years in some cases [73]. In addition, improvements in processing speed were related to improvements in everyday functioning, including instrumental activities of daily living and driving abilities, providing evidence of far-transfer effects [73]. Importantly, one of the biggest predictors of cognitive gains following processing speed training is pre-training processing speed abilities, such that persons with slower initial speed benefit the most from training [73].

One recent study examined the effects of processing speed training in young, healthy adults [74]. This research group found that processing speed training (using several adaptive training tasks, such as a Visual Number Matching task) led to near-transfer improvements in processing speed in adults with a mean age of 21.6 years, although no evidence of far-transfer was found in measures of fluid intelligence, working memory, or inhibition [74]. Another group found increases in efficiency of attentional resource allocation, as measured by pupil dilation, following processing speed training in young adults [75]. Thus, there are mixed results on near- and far-transfer gains of processing speed training with healthy populations, with evidence suggesting that initial processing speed abilities are an important moderator in treatment outcomes.

Notably, some researchers have examined processing speed training as an active control condition in cognitive remediation studies (for example, compared to working memory training) [76], due to weak evidence of transfer effects, particularly far-transfer, associated with processing speed training in healthy adults [74]. However, a closer look at the literature suggests that individuals with lower initial processing speed (for example, older adults) can benefit from processing speed training, including both near-transfer effects and far-transfer effects (for example, improvements in activities of daily living) [73].

#### Schizophrenia patients

Given that schizophrenia patients have prominent deficits in processing speed and that processing speed has been strongly related to everyday functioning, it logically follows that processing speed training may be an important method of improving functioning in patients. Examinations of processing speed training with schizophrenia patients to date have solely been part of larger programs of cognitive remediation, with several studies finding that broad-based cognitive remediation programs lead to improvements in processing speed in patients [77–80]. Overall, a recent meta-analysis shows a medium-sized effect (*d* = .48) of general cognitive remediation therapy on improving processing speed across a total of 655 schizophrenia patients [4]. However, targeted processing speed training studies with schizophrenia patients are needed to better understand the mechanisms of processing speed enhancement through training in patients. Given that individuals with slower initial speed appear to benefit the most from training, coupled with the fact that schizophrenia patients typically have reduced processing speed abilities (that is, approximately 1 to 2 standard deviations below healthy controls) [81], the current study conceptualizes processing speed training as an active training condition rather than an active control condition for individuals with schizophrenia.

### Objectives and hypotheses

This study will investigate 1) near-transfer gains associated with working memory and processing speed training in schizophrenia patients (that is, gains in the working memory or processing speed domains, respectively); 2) far-transfer gains associated with working memory and processing speed training in schizophrenia patients (that is, gains in other neurocognitive domains, primarily fluid intelligence, and gains in social cognition); and 3) real-world gains associated with working memory and processing speed training in schizophrenia patients (that is, gains in daily functioning). In addition, exploratory analyses will examine the effect of individual difference variables (that is, sleep quality and intrinsic motivation) on training-related gains. The primary outcome measures are as follows: working memory, processing speed, and fluid intelligence.

The corresponding hypotheses are as follows: 1) post-training performance in working memory and processing speed will improve in the working memory training group and processing speed training group, respectively, relative to the no-training control group (that is, both training groups will show near transfer in their own domain); 2) both working memory and processing speed training will lead to more generalized improvements in other neurocognitive domains, particularly fluid intelligence, compared to the no-training control group, as well as enhancements in social cognition relative to the no-training control group; and 3) both working memory and processing speed training will lead to more improvements in daily functioning compared to the no-training control group. Given the current paucity of research comparing the gains associated with specific training programs, no hypotheses are made with regards to which training group (that is, working memory or processing speed) will show greater improvements in neurocognition, social cognition, or daily functioning. Rather, comparisons between training groups will be exploratory in nature.

## Methods/Design

### Study design

This study is a randomized controlled trial (RCT) and will be conducted at the Foothills Medical Centre and the University of Calgary in Calgary, Alberta, Canada. Participants who meet eligibility criteria will be randomly allocated to a working memory-training group, a processing speed-training group, or a no-training control group. The study will employ a parallel design with a 1:1:1 allocation ratio. Participants allocated to the working memory-training group or processing speed-training group will directly receive the specified cognitive training intervention, whereas those allocated to the no-training control group will be given the opportunity to receive one of the two cognitive training interventions after a waiting period of 10 weeks. Assessments are planned both before and after the intervention for the experimental groups, and both before and after the 10-week wait period for the control group.

### Recruitment process and study population

Up to 81 schizophrenia patients will be recruited. This is based on a power calculation with an alpha-level of .05, power of .80, and an effect size of *d* = .70, with the effect size derived from a recent study examining a more specific cognitive remediation program (that is, auditory working memory training) in schizophrenia [58]. Importantly, this sample size accounts for dropout rates of up to 10 %, based on previous cognitive training studies [82]. Recruitment status is currently pending, with plans to start in January 2016. Patients will be recruited through outpatient clinics at Foothills Medical Centre and through community support programs in Calgary, Alberta, after receiving appropriate permissions to enter the clinics and discuss the study. Participants will also be recruited through an existing participant database at the University of Calgary. Inclusion criteria for all participants will be as follows: (1) a diagnosis of schizophrenia or schizoaffective disorder, assessed with the Structured Clinical Interview for DSM-5 (SCID-5) interview; (2) age 18 to 65 years; (3) no uncorrected visual impairment, including color blindness, determined by self-report; (4) no uncorrected hearing impairment, determined by self-report; and (5) able to provide informed consent, determined by the clinical experience of the researchers. Exclusion criteria will be as follows: (1) meeting Diagnostic and Statistical Manual - 5^th^ edition (DSM-5) diagnostic criteria for a current major depressive episode, manic episode, or hypomanic episode, assessed with the SCID-5 interview; (2) use of electroconvulsive therapy (ECT) or transcranial magnetic stimulation therapy (TMS) within the past month, determined by self-report; (3) recent (that is, within past 3 months) history of substance-use disorder (excluding nicotine, cannabis, or caffeine), assessed with the SCID-5 interview; (4) diagnosed with a medical condition known to affect cognition (for example, endocrine disease or uncontrolled diabetes), determined by self-report; and (5) score less than 70 on the Wechsler Abbreviated Scale of Intelligence (WASI-II). In addition, only individuals who have access to the internet on a home computer will be eligible to participate in the study.

**Fig. 1:**
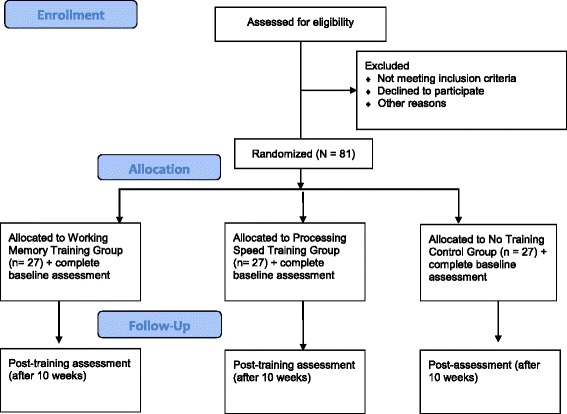
Participant flow through study. This figure illustrates participant enrollment, allocation, and follow-up throughout the study

See Fig. 1 for participant flow chart. Participants who are interested will sign a “consent to contact” form and will be contacted by the research team via e-mail and/or telephone, or participants can contact the study investigators themselves through the provided e-mail and/or telephone number. Interested participants will undergo a telephone screening process to ensure that they meet the aforementioned inclusion criteria. Those who meet eligibility criteria will be invited for the first baseline assessment (see full battery below), which will include confirming clinical diagnoses with the SCID-5 Research Version. Participants whose schizophrenia or schizoaffective disorder diagnosis is confirmed with the SCID-5 will be invited for the second baseline assessment, which will include pre-training cognitive assessment (see full battery below). Participants will be instructed to continue taking their medication throughout study participation, and information regarding medication usage and dosage will be collected at pre- and post-assessments.

All participants who meet the inclusion criteria will be randomly assigned to one of three conditions: (1) a working memory-training condition (27 participants), (2) a processing speed-training condition (27 participants), or (3) a no-training control condition (27 participants). Participants in the two training conditions will be blind to the fact that there are multiple training groups and to which cognitive domain they have been assigned to (that is, no mention of “working memory” or “processing speed” will be made). Group assignment will be determined by a Microsoft Excel randomization generator (that is, computer-generated random numbers). The random allocation sequence will be generated by a trained research assistant. Randomization will be performed as complete randomization with a 1:1:1 allocation. The allocation sequence will be concealed in sealed envelopes until the end of the pre-training assessment. A trained research assistant will also assign participants to interventions. Participants who are randomly assigned to the no training control condition will be given the opportunity to access the cognitive training games after their post-assessments. All researchers administering pre- and post-assessments will be blind to the group assignments of each participant.

### Concurrent therapy

All participants will be maintained on the same medications throughout the entire study period, as medically feasible, with no introduction of new chronic therapies. Data will not be analyzed from participants who make changes to the type of treatment they are receiving during the 10-week study period. However, data from participants who change the dose of their medication, without changing the type, will be analyzed. Standard therapy (for example, medications and psychotherapy) for schizophrenia is allowed, except for any other cognitive training games.

### Ethics, consent, and permissions

This study has been reviewed and approved by the Conjoint Health Research Ethics Board (CHREB) at the University of Calgary (study ID number: REB15-0526). The study has been registered by ClinicalTrials.gov (registration number: NCT02478827). All participants will receive verbal and written information regarding the study procedures, and all participants must provide written informed consent before inclusion in the study. This will be completed by graduate students and/or trained research assistants. Any protocol amendments will be reported immediately to the ethics board and will be discussed in the final manuscript. All data will be kept in password protected digital files with individual names removed to ensure confidentiality.

### Discontinuation of participants

Participants will be discontinued from the study if they withdraw their consent. Participants may be withdrawn from the study if they experience a significant increase in schizophrenia symptoms.

### Training programs

Processing speed and working memory-training programs will be provided by BrainGymmer [83] and accessed online. BrainGymmer was chosen over other training programs due to its interesting and engaging user interface, engaging exercises, and flexibility in game design (that is, games were easily adapted to incorporate researcher ideas). Moreover, the BrainGymmer program is relatively inexpensive compared to other programs and therefore may be affordable for this population.

All participants will be instructed to train for 30 minutes per day, 5 days per week, for a total of 10 weeks in a comfortable location of their choice (for example, home computer). This time frame (that is, 25 hours of training) was chosen based on research indicating that trained skills improved substantially during the first 20 to 25 hours of cognitive training and only minimally thereafter in individuals at high risk for psychosis [84]. Each training program will consist of three exercises (described below), and participants will be instructed to distribute their time approximately equally across the three exercises. Notably, all exercises are adapted to individual performance, such that the difficulty level is increased if the individual’s performance is above the threshold for their current level, and difficulty level is decreased if the individual’s performance falls below the minimum performance required at the current level. Difficulty thresholds on BrainGymmer were determined based on internal play testing, test panel feedback (with members from over 20 different countries), user feedback, and game result data. Training compliance will be monitored through weekly electronic data upload, and participants will receive phone call reminders if compliance is low (that is, below 50 % of the required training for the week).

#### Working memory training

Participants who are randomly assigned to receive working memory training will be instructed to access the following three games: *N Back*, *Multi Memory*, and *Moving Memory*. All three exercises incorporate both maintenance and manipulation aspects of working memory. Examples of these exercises can be seen in Additional file 1.

In *N Back*, cards with various shapes and colors appear on the screen and participants are asked to remember the shapes and colors. The goal of the exercise is to determine whether the current card matches with the card that was shown *n*-back cards before the current one. The difficulty level is increased by increasing the number of cards between the current stimulus and the one it is being compared to (that is, the “*n*”).

In *Multi Memory*, tiles appear in a grid and participants are asked to remember the shape, color, and position of the tiles to try to reproduce them after the display disappears. The level of difficulty is increased by increasing the number of tiles, the size of the grid, the number of different shapes, and the length of time the user is given to look at the tiles.

Finally, in *Moving Memory*, tiles with colored shapes appear on the screen, and each tile has a unique number written below. Participants are required to remember the numbers associated with each colored shape to allow them to choose a pair of numbers later that are associated with matching shapes. Each time a correct matching pair is identified, the tiles move to new positions, thus requiring participants to use the numbers, rather than spatial locations, to correctly match shape-pairs. Difficulty level is increased by increasing the number of card pairs to be matched, increasing the number of background colors, shapes, and shape colors, and by decreasing the amount of time the display is shown.

#### Processing speed training

Participants who are randomly assigned to receive processing speed training will be instructed to access the following three games: *Line It Up*, *Sliding Search*, and *Bubble Math*. All three exercises require timely information processing and have a minimal memory component. Examples of these exercises can be seen in Additional file 2.

In *Line It Up*, colored tiles with different shapes appear on the screen in a horizontal line (that is, reference line). Participants are required to order a shorter line of colored tiles (that is, target line) using the first line as a reference as quickly as possible. The target line contains only a portion of the tiles present in the reference line. Difficulty level is increased by increasing the lengths of the target and reference lines, with shapes and colors that begin to look very similar.

In *Sliding Search*, a grid of several reference images is presented at the top of the screen, and participants are required to choose which image matches the image shown at the bottom of the screen as quickly as possible. Difficulty is increased by increasing the speed at which the image is moved across the bottom of the screen (that is, requiring faster responding), as well as changing the reference grid to include more similar, nuanced images.

In *Bubble Math*, participants are asked to complete simple math equations, which are moving across the screen, as quickly as possible. Difficulty is increased by increasing the number of questions asked per minute as well as the range of numbers used in the problems.

### Outcome measures

#### Baseline measures

At baseline, participants will complete a form assessing demographic factors (sex, age, ethnicity, income, education, and maternal and paternal education). Next, the Vocabulary and Matrix Reasoning subtests of the WASI-II [85] will be used to estimate the IQ of all participants. Participants will then go through the SCID-5 for DSM-5 semi-structured interview. Finally, the Positive and Negative Syndrome Scale (PANSS) [86] will be used to assess symptom severity in all participants.

#### Pre-training and post-training measures

The following neurocognitive, social cognitive, and functional status measures will be administered prior to randomization into training, as well as immediately post-training. The entire battery will be approximately 2 to 4 hours in duration at each sitting (see Table 1).

**Table 1:** List of all tasks and measures

Task	Cognitive domain	Primary or secondary outcome	Transfer hypothesis (WM)	Transfer hypothesis (PS)	Pre-test time	Post-test time
Demographic information form	N/A	N/A	N/A	N/A	10 minutes	N/A
WASI-II Vocabulary	Crystallized intelligence	N/A	N/A	N/A	15 minutes	N/A
WASI-II Matrix Reasoning	Fluid intelligence	N/A	N/A	N/A		
SCID-5-RV for DSM-5	N/A	N/A	N/A	N/A	2 h 30 min	N/A
PANSS	N/A	Secondary	N/A	N/A	40 minutes	40 minutes
Session 1 total:					3 h 35 min	
Neurocognition measures:						
N-Back Task	Working memory (visual)	Primary	Near	Far	15 minutes	15 minutes
WAIS-IV Digit Span	Working memory (auditory)	Primary	Near	Far	5 minutes	5 minutes
Maintenance and manipulation (computer)	Working memory (visual-spatial)	Primary	Near	Far	15 minutes	15 minutes
WAIS-IV Symbol Search	Processing speed	Primary	Far	Near	5 minutes	5 minutes
D-KEFS Color Naming	Processing speed	Primary	Far	Near	N/A	N/A
D-KEFS Color-Word Interference Test	EF: Inhibition, set-shifting	Secondary	Far	Far	5 minutes	5 minutes
D-KEFS Trail Making Test	EF: Cognitive flexibility	Secondary	Far	Far	5 minutes	5 minutes
Raven’s Standard Progressive Matrices	Fluid intelligence	Primary	Far	Far	25 minutes	25 minutes
Cattell’s Culture Fair Test	Fluid intelligence	Primary	Far	Far	15 minutes	15 minutes
Social cognition measures:						
Geneva Emotion Recognition Test	Emotion recognition	Secondary	Far	Far	20 minutes	20 minutes
Hinting task	Theory of mind	Secondary	Far	Far	10 minutes	10 minutes
Beliefs regarding cognition measures:						
Need for Cognition Scale	N/A	Secondary	Far	Far	10 minutes	10 minutes
Theories of Intelligence Scale	N/A	Secondary	Far	Far	5 minutes	5 minutes
Daily functioning measures:						
Cognitive Failures Questionnaire	N/A	Secondary	Far	Far	5 minutes	5 minutes
UPSA-Brief	N/A	Secondary	Far	Far	15 minutes	15 minutes
SOFAS	N/A	Secondary	Far	Far	N/A	N/A
Questionnaires:						
Brief Sleep Questionnaire	N/A	N/A	N/A	N/A	5 minutes	5 minutes
Motivation Form	N/A	N/A	N/A	N/A	5 minutes	5 minutes
Pre- and post-test total:					2 hr 45 min	3 hr 30 min

#### Neurocognitive measures

Neurocognitive measures will examine working memory, processing speed, executive functions, and fluid intelligence.

##### Working memory - N-Back task

A participant’s ability to maintain, monitor, and manipulate information will be assessed with an N-Back task. In this task, participants will view a set of visually presented random shapes and will indicate whether each stimulus matches the stimulus that appeared *n* trials previously (2-, 3-, or 4-back). This task has been used as a measure of working memory in previous studies [33]. This task will be used as a near-transfer measure of working memory.

##### Working memory - Digit Span Task

Auditory working memory abilities will be assessed using the Digit Span subtest of the Wechsler Adult Intelligence Scale 4^th^ Edition (WAIS-IV) [65]. In this task, participants will first be required to repeat verbally presented digits in the same order that they were presented (digits forward), then in reverse order (digits backwards), and finally in numerical order (digits sequencing). Digit Span is one of the most widely used measures of working memory, and assesses both maintenance and manipulation aspects of working memory [87].

##### Working memory - Spatial Maintenance and Manipulation Task

Visuospatial working memory will be assessed using the computerized Spatial Maintenance and Manipulation task [88]. In the spatial maintenance condition, participants are asked to remember the positions of circles on the screen after a short delay. In the maintenance and manipulation condition, participants are asked to mentally rotate the positions of the circles across a horizontal plane and remember the new position of the circles. This talk allows for an examination of the different components of working memory, with the maintenance condition assessing short-term memory and the manipulation condition examining also the executive aspects of working memory.

##### Processing speed - Symbol Search Test

To examine processing speed abilities, two tasks will be used. First, the Symbol Search subtest of the WAIS-IV will require participants to quickly identify symbols that match one of two target symbols. This test is considered the most pure processing speed test available [65].

##### Processing speed - Color Naming Task

In addition, the Color Naming condition of the Color-Word Interference Test on the Delis-Kaplan Executive Function System (D-KEFS) [89] will be administered. This task requires participants to name the color of patches of ink across a page (red, blue, or green) as quickly as possible. This task has a low working memory loading and provides a measure of verbal processing speed [90].

##### Inhibitory control and set shifting - Color-word Interference Test

To examine executive functions (EFs), two full subtests of the D-KEFS will be administered. First, the Color-word Interference Test (CWIT) from the D-KEFS [89], which is based on the classic Stroop test, will be used as a measure of inhibitory control and set-shifting abilities. One condition of this test requires participants to name the color of the ink in which the words are printed while inhibiting the more salient response of reading the word. In another condition, participants are asked to switch between naming the color of the ink in which words are printed and reading the word aloud while not naming the ink color.

##### Cognitive flexibility - Trail Making Test

Another EF, cognitive flexibility, will be examined with the Number-Letter Switching Condition of the Trail Making Test [89]. In this visual-motor sequencing task, participants are required to alternate between connecting numbers and letters in ascending order.

##### Fluid intelligence - Raven’s standard progressive matrices

The Raven’s Standard Progressive Matrices [91] will be used as a measure of fluid intelligence. The test will be split into parallel even and odd forms of 30 items each and will be administered in a counterbalanced fashion across participants at pre- and post-training. In this task, participants are presented with a matrix of figures where one of the figures is empty. Through deducing the relationships between columns and rows, the participant is required to infer which figure should be placed in the empty position from six possible response options. The Raven’s test has been used to demonstrate gains in fluid intelligence following working memory training [31]. It is also one of the most widely used measures of fluid intelligence.

##### Fluid intelligence – Cattell’s Culture Fair Test

The Cattell’s Culture Fair Test (CCFT) Scale 3 will also be used as a measure of fluid intelligence [92]. Forms A and B will be administered in a counter-balanced fashion across participants at pre- and post-test. CCFT Scale 3 contains four subtests, including series, classifications, matrices, and conditions. The CCFT is considered a more comprehensive measure of fluid intelligence than matrix only tasks [93].

#### Social cognitive measures

Social cognitive measures will examine emotion recognition and theory of mind abilities.

##### Emotion recognition - Geneva Emotion Recognition Test

First, the Geneva Emotion Recognition Test will be used to examine emotion recognition abilities using face, voice, and body cues. This test includes 83 short video clips with sound, with 10 actors portraying 14 different emotions. Following each clip, participants are asked to choose which of 14 emotions the actor was trying to portray [94]. Importantly, this task also requires timely information processing due to the brief nature of the clips.

##### Theory of mind - Hinting Task

Finally, the Hinting Task, one of the few consistently utilized theory of mind measures in the schizophrenia literature, will be administered. This task requires participants to listen to a verbally presented story and identify the intention of one character when he or she provides a verbal hint to a second character. The 10 items from the original Hinting Task [95], as well as the 10 more recently developed scenarios [96] will be utilized, with five questions from each set being randomly selected and administered at pre-assessment, and the other five questions from each set administered at post-assessment.

#### Beliefs regarding cognition

The *Need for Cognition Scale* and the *Theories of Intelligence Scale* will be used to examine participants’ beliefs regarding cognition.

##### Need for cognition scale

The short form of the Need for Cognition Scale [97] will be used to examine how much participants enjoy cognitively stimulating or challenging tasks. Responses to this questionnaire are provided on a 5-point Likert scale with 18 questions in total.

##### Theories of Intelligence Scale

The Theories of Intelligence Scale is an eight-item scale that will be used to examine the degree to which participants believe that cognitive abilities (that is, intelligence) are fixed or malleable [98]. Responses to this questionnaire are provided on a 6-point Likert scale.

#### Measures of functional status

Cognitive, social, and occupational functioning in daily life will be examined.

##### Functioning - Cognitive Failures Questionnaire

The Cognitive Failures Questionnaire (CFQ) will assess an individual’s proneness to cognitive errors and slips while completing everyday tasks. This measure has been successfully utilized with schizophrenia patients to examine perception, memory, and motor lapses in daily life [99].

##### Functioning - UCSD Performance-based Skills Assessment - Brief

The UCSD Performance-Based Skills Assessment (UPSA-Brief) [100] will be used to assess functional capacity in schizophrenia patients. This role-play test will be used to assess performance in two basic living skills: finance and communication.

##### Functioning – social and occupational functioning assessment scale

In addition, the Social and Occupational Functioning Assessment Scale (SOFAS) [101] will be used to assess social and occupational functioning using a clinician-rated scale between 1 and 100.

#### Questionnaires

Two questionnaires, related to sleep and motivation, will be administered to each participant.

##### Brief sleep questionnaire

Given that sleep quality has been associated with cognitive functioning, a brief measure will be administered. All patients will be asked the following questions based on the previous month: 1) What time do they usually go to bed? 2) How long does it usually take them to fall asleep? 3) What time do they usually wake up? and 4) How many hours of actual sleep do they usually obtain each day? These questions were adapted from the Pittsburgh Sleep Quality Questionnaire [102].

##### Intrinsic motivation inventory

Finally, brief self-report motivation questionnaires (that is, the Intrinsic Motivation Inventory) [103] will be provided to patients to complete prior to training, during training (that is, one time approximately half-way through training), and immediately post-training. This questionnaire will measure motivation, engagement, and self-regulation.

### Procedure

In the first session, following informed consent (consent form provided in Additional file 3), demographic information will be collected and the Vocabulary and Matrix Reasoning subtests of the WASI-II will be administered by trained graduate students. Next, the trained students will interview participants with the SCID-5. Finally, the PANSS will be administered. The entire first session should last approximately 3 hours and 35 minutes.

The second session will include pre-assessment of all neurocognitive, social cognitive, and functioning measures described above. This session will be approximately 2 hours and 45 minutes in duration. To reduce the impact of fatigue and carryover effects on the results, the administration order of all cognitive and functional measures will be counter-balanced using a Williams Design Latin Square [104]. Testing will be administered by trained graduate students and research assistants, who will be blind to the randomization until completion of the baseline assessment. Blinding will only be broken if knowledge of the patient’s treatment group is necessary for further patient management. Following the baseline assessment, participants will be randomized and provided with training instructions, including how to access the online training in addition to the training schedule. Training program names (for example, *Bubble Math*, *Moving Memory*) will not be concealed; however, participants will not be informed of the objectives or hypotheses of the current study, and will be unaware of the other training conditions. Participants will be contacted half way through training (after approximately 5 weeks) to complete the mid-point motivation questionnaire.

The 3 hour 30 minute post-training assessment will be composed of the same measures (that is, neurocognitive measures, social cognitive measures, and measures of functional status) described above and using parallel forms where indicated, in addition to a re-administration of the PANSS. All post-assessments will be conducted by trained graduate students and research assistants who are blind to the randomization of each participant. All assessment materials will be scripted to ensure consistency across examiners and across assessments.

### Adverse events

Participants will be asked to provide personal information about their own and their family history of mental and medical disorders. Revealing this personal information may lead to discomfort or may evoke unpleasant memories and/or anxiety. If a participant becomes upset during the diagnostic interview, the researcher will stop, provide support, and provide the participant the chance to decide whether they want to discontinue, reschedule, or continue. The interview will be conducted by trained research assistants or graduate students, who will appropriately deal with any anxiety or stress related to the interview. Community resources will be provided to individuals who are distressed and would like to talk to someone about the experiences that have been brought up in the interview.

In recognition of the high level of concentration needed to complete a single session of training and the commitment needed to sustain training over several weeks, we have decided to keep the training to absolute minimal levels for which the literature has indicated effects. Beyond keeping the cognitive demands from training to a minimum, we will be careful to protect participants emotionally by not exposing them to how their performance on any of the tasks relates to that of others. Due to the nature of adaptive training tasks, participants will be able to tell if their performance has improved session by session; however, no explicit feedback will be provided by the researchers. Participants are allowed to discontinue participation in the study at any point without any penalty to them.

### Data sets analyzed

All eligible participants who are randomized into the study and complete at least 50 % of the cognitive training will be included in the completer analysis, following similar cut-offs in prior cognitive training research [Lawlor-Savage L, Goghari VM: Dual n-back working memory training in healthy adults: a randomized comparison to processing speed training, submitted]. All eligible participants who are randomized into the study will be included in the intent-to-treat analysis. Data collection will stop when the goal sample size has been reached or when new participants are unable to be recruited for a significant period.

### Statistical analysis

The following demographic variables at screening will be summarized: ethnicity, sex, age, and education. Between-group analyses of variance (ANOVAs) and chi square analyses will be used to examine any baseline differences between groups in demographics, individual characteristics (for example, sleep), cognition, and/or functioning. Any baseline differences will be corrected in all further analyses as appropriate.

Next, a three-group (working memory training, processing speed training, or no training) x time (pre-training or post-training) repeated measures ANOVA will be conducted to test for significant group differences in each domain of neurocognition (that is, memory, processing speed, executive functioning, and fluid intelligence) over time. Follow-up one-way repeated measures ANOVAs will be conducted as necessary to further examine the main effects of time. Follow-up independent *t*-tests will be conducted to examine differences in training- related gains between each training group and the no-training control group, based on the above-described *a priori* study objectives. Cohen’s *d* effect sizes will be reported. Notably, composite measures will be used where applicable (for example, a working memory composite, a processing speed composite).

Similarly, a group x time repeated measures ANOVA will be conducted to test for significant group differences in each social cognition task and each measure of daily functioning, with follow-up one-way repeated measures ANOVAs to follow-up on main effects of time and independent *t*-tests to examine group differences. Again, Cohen’s *d* effect sizes will be reported.

Finally, exploratory simple regression analyses will be conducted to examine potential moderating factors of training-related change. For any aspects of cognition and functioning that show significant training-related gains, regression analyses will be conducted using individual difference variables (for example, medication usage, sleep quality, duration of illness, intrinsic motivation) as the independent variable and change scores for each cognitive or functional domain as the dependent variable.

### Publications

The final study results will be submitted as a manuscript for publication. No restrictions on publication exist. This information will be made available to the recruitment clinics and study participants.

## Discussion

The results of the current study will shed light on whether targeted cognitive training programs can improve not only cognition, but also daily functioning in the lives of schizophrenia patients. Given the recently widespread availability of “brain training” games on the Internet, the benefits of cognitive training for individuals with schizophrenia should be examined to identify whether these games can be recommended as part of an intervention program. Overall, the current study may benefit individuals with schizophrenia who want to enhance their cognition.

The strengths of the current study include that two more specific training program are being utilized, which will provide more specific information on the mechanisms of training-related gains than the broad-based training programs that have been used in the previous literature. Additionally, the current study is employing multiple measures to assess each cognitive domain and thus will provide a more comprehensive analysis of training gains. Both a strength and limitation of the current study is the number of outcome measures that are being utilized. This is a strength of the design, given that it allows us to thoroughly examine many different near- and far-transfer effects associated with working memory and processing speed training in schizophrenia. However, this may also be viewed as a limitation of the current design, as it may lead to errors associated with multiple comparisons. To address this issue, composite measures will be used in the statistical analyses where applicable (for example, a working memory composite). In addition, two primary outcome measures have been chosen for each group, as described above (that is, working memory composite and fluid intelligence composite for the working memory group, and processing speed composite and fluid intelligence composite for the processing speed group). Finally, a limitation with this line of research is the difficulty in obtaining pure measures and pure training programs of each cognitive domain. To address this issue, we have included multiple assessment measures of each cognitive domain. With regards to the training programs, we recognize the difficulty in creating games that solely train working memory abilities and processing speed abilities, though we are confident that the selected games predominantly train the cognitive domain of interest. However, we will also examine potential gains in other domains, as described above.

### Trial status

The current trial has received ethics approval from the University of Calgary Conjoint Health Research Ethics Board (CHREB). Recruitment of study participants has not yet commenced but is tentatively scheduled to begin in January 2016.

## Additional files

Additional file 1:
**Examples of Working Memory Training games provided by BrainGymmer.** (DOC 299 kb) Additional file 2:
**Examples of Processing Speed Training games provided by BrainGymmer.** (DOC 683 kb) Additional file 3:
**Consent form to be provided to all participants.** (DOC 54 kb)

## Abbreviations

ANOVA: analysis of variance
CCFT: Cattell’s Culture Fair Test
CFQ: Cognitive Failures Questionnaire
CHREB: Conjoint Health Research Ethics Board
CWIT: Color-word Interference Test
D-KEFS: Delis-Kaplan Executive Function System
DSM-5: Diagnostic and Statistical Manual - 5^th^ edition
ECT: electroconvulsive therapy
EF: executive function
PANSS: Positive and Negative Syndrome Scale
RCT: randomized controlled trial
SCID-5: structured clinical interview for the DSM-5
SOFAS: Social and Occupational Functioning Assessment Scale
TMS: transcranial magnetic stimulation
UPSA: UCSD Performance-based Skills Assessment
WAIS-IV: Wechsler Adult Intelligence Scale – 4^th^ Edition
WASI-II: Wechsler Abbreviated Scale of Intelligence – 2^nd^ Edition
