# Supplementary material for: Working memory and processing speed training in schizophrenia: study protocol for a randomized controlled trial
Source: Trials. 2016 Jan 26;17:49. doi: 10.1186/s13063-016-1188-5 (PMC4728776; doi:10.1186/s13063-016-1188-5)

**Additional file 1**

Screen shots of *N Back* game:


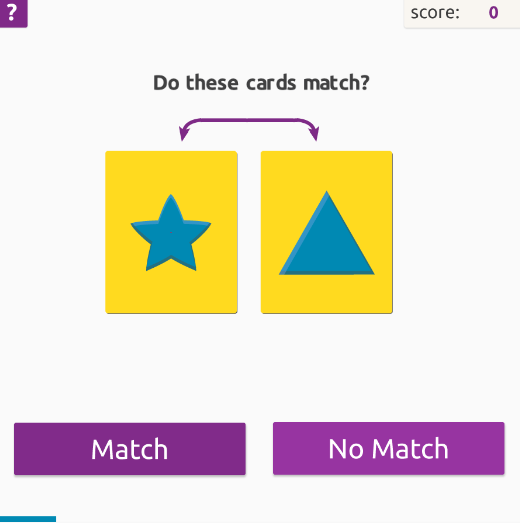

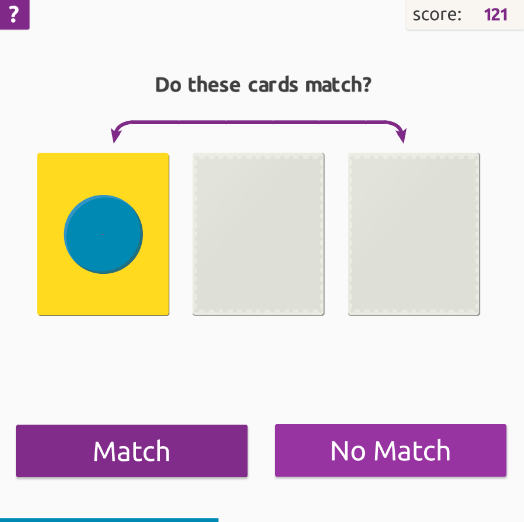


Screen shots of *Multi Memory* game:


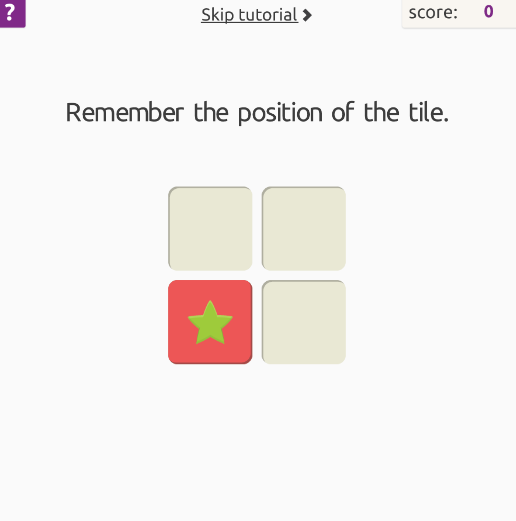

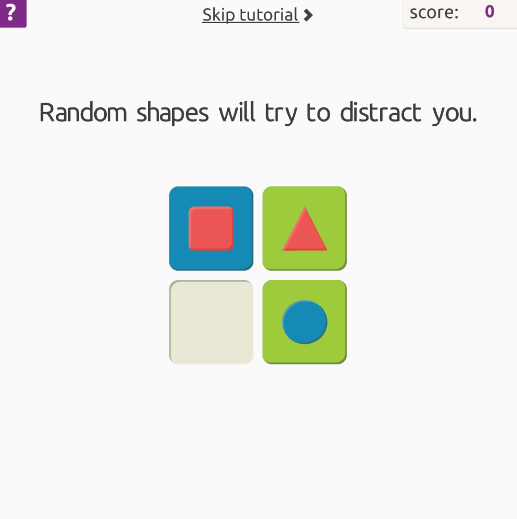

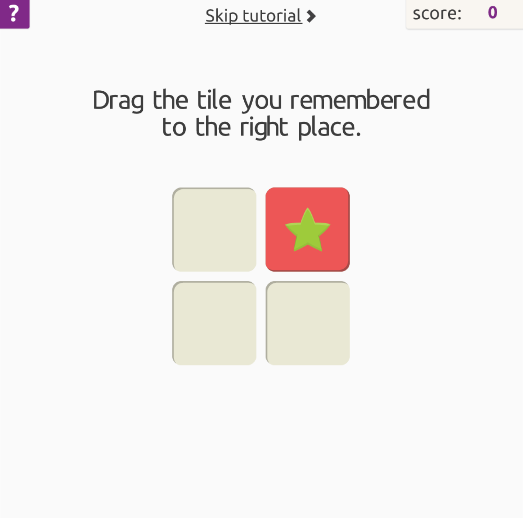


Screen shots of *Moving Memory* game:


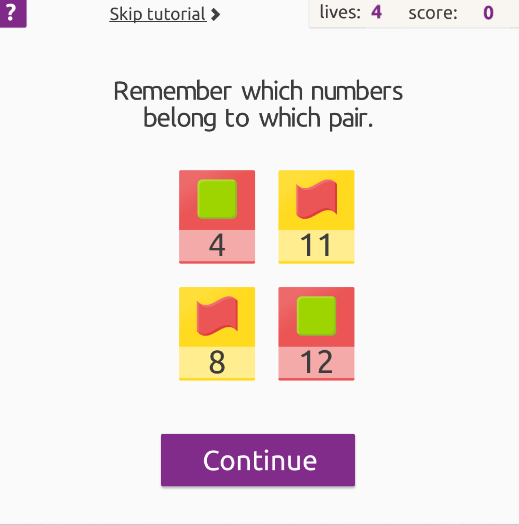

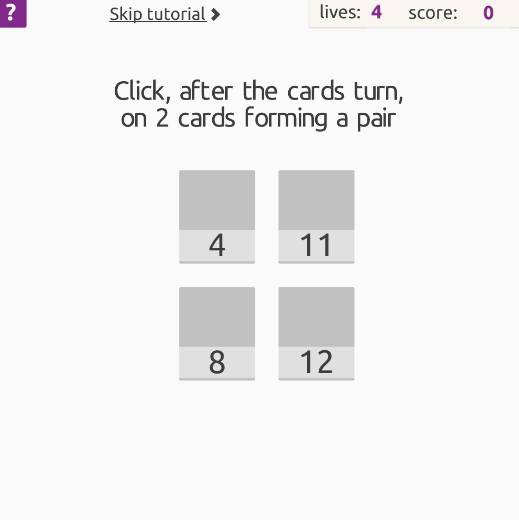

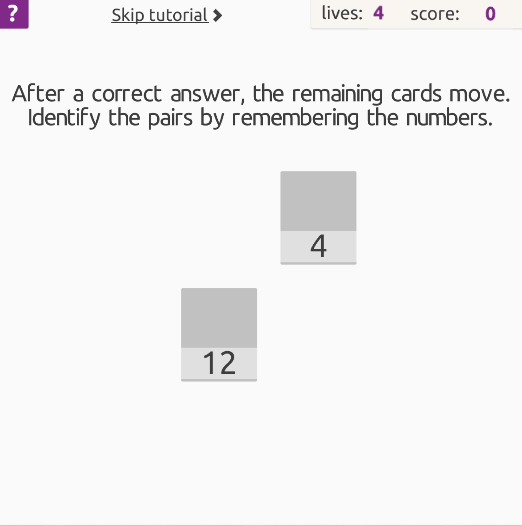

Supplement: Additional file 1: — Examples of Working Memory Training games provided by BrainGymmer. (DOC 299 kb) [file 13063_2016_1188_MOESM1_ESM.doc]
