# Supplementary material for: Working memory and processing speed training in schizophrenia: study protocol for a randomized controlled trial
Source: Trials. 2016 Jan 26;17:49. doi: 10.1186/s13063-016-1188-5 (PMC4728776; doi:10.1186/s13063-016-1188-5)

**Additional file 2**

Screen shots of *Line It Up* game:


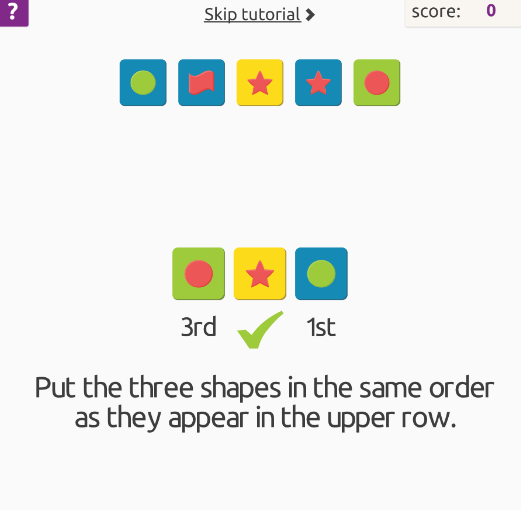

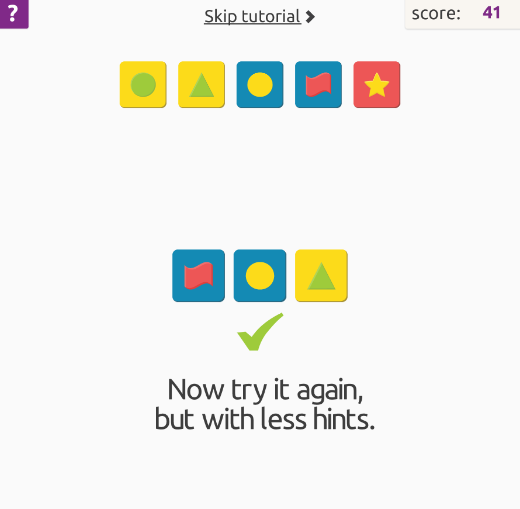

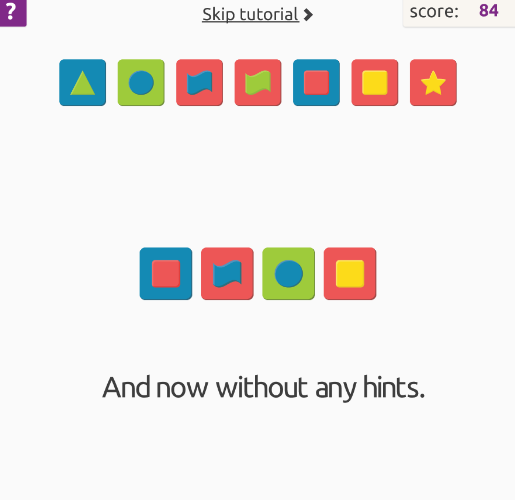


Screen shots of *Sliding Search* game:


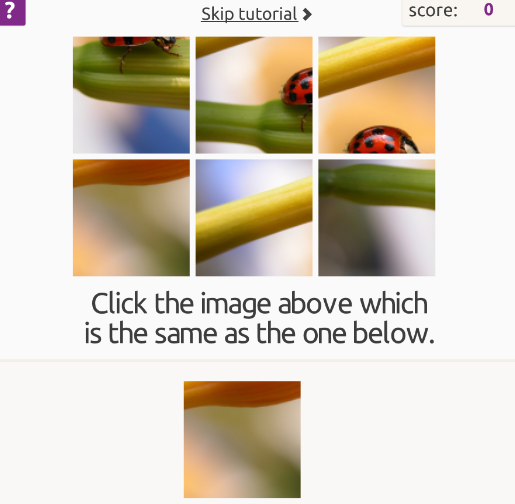

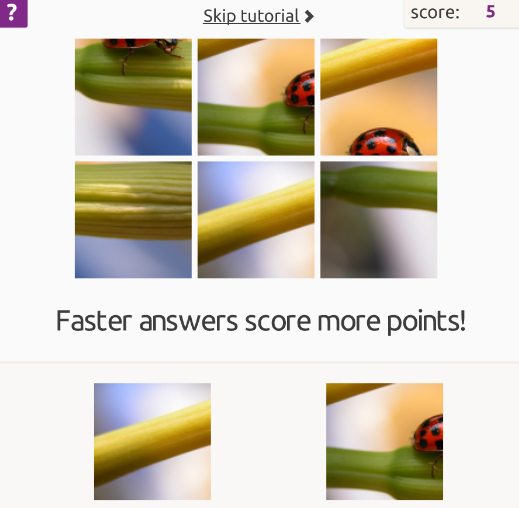


Screen shots of *Bubble Math* game:


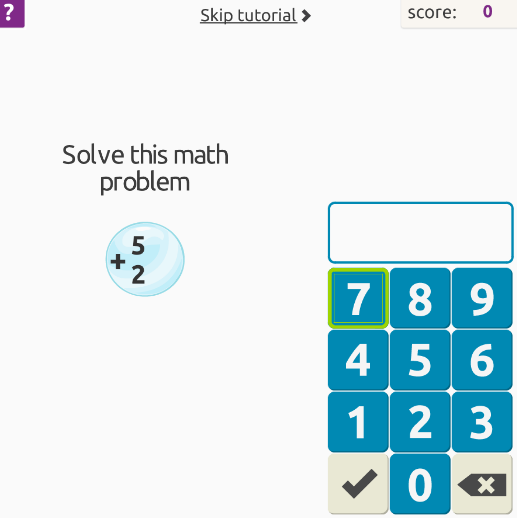

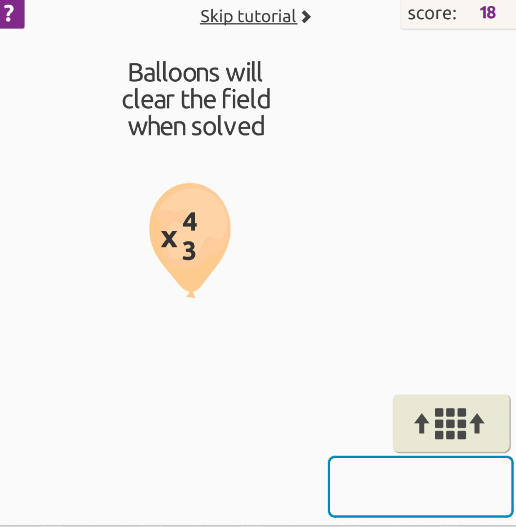

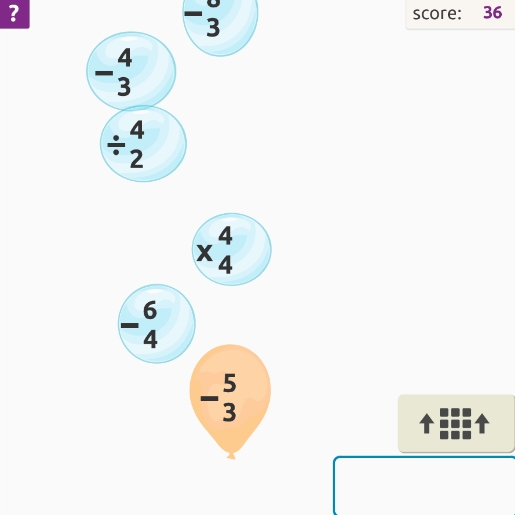

Supplement: Additional file 2: — Examples of Processing Speed Training games provided by BrainGymmer. (DOC 683 kb) [file 13063_2016_1188_MOESM2_ESM.doc]
