# Supplementary material for: Working memory and processing speed training in schizophrenia: study protocol for a randomized controlled trial
Source: Trials. 2016 Jan 26;17:49. doi: 10.1186/s13063-016-1188-5 (PMC4728776; doi:10.1186/s13063-016-1188-5)
Supplement: Additional file 3: — Consent form to be provided to all participants. (DOC 54 kb) [file 13063_2016_1188_MOESM3_ESM.doc]

**Appendix C**


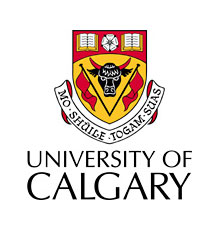
Consent Form

**DEPARTMENT OF PSYCHOLOGY**

Administration Building, Room 214

www.psych.ucalgary.ca

Telephone: 403-210-7344

Fax: 403-282- 8249

**TITLE**: COGNITIVE TRAINING IN SCHIZOPHRENIA

**SPONSOR:** University of Calgary

**RESEARCHER:** Briana Cassetta, Department of Psychology, 587-225-2443, bcassett@ucalgary.ca

**SUPERVISOR:** Dr. Vina Goghari, Departments of Psychology and Psychiatry, vina.m.goghari@ucalgary.ca

This consent form is only part of the process of informed consent. It should give you the basic idea of what the research is about and what your participation will involve. If you would like more detail about something mentioned here, or information not included here, please ask. Take the time to read this carefully and to understand any accompanying information. You will receive a copy of this form.

You do not have to take part in this study and your care does not depend on whether or not you take part. This study is being conducted by members of the Department of Psychology and Psychiatry at the University of Calgary.

**Your participation in this study is entirely voluntary. Please take your time to make your decision.**

**“WHY IS THIS STUDY BEING DONE?”**

The purpose of this study is to test a cognitive training program to study the effects of training on cognitive, emotional, clinical, and daily functioning outcomes in people with schizophrenia compared to controls.

**“WHAT DO WE HOPE TO LEARN?”**

We hope to find out whether people with schizophrenia will benefit from a cognitive training program. We hope that this information could help improve cognitive and daily functioning in those with schizophrenia and help facilitate the development of treatments for these individuals.

The specific objectives of this study include:

1. To evaluate the effects of a 10-week cognitive training program in people with schizophrenia.

**“WHAT IS INVOLVED IN THIS STUDY?”**

If you choose to participate, you may be asked to complete:

1. A clinical research interview that will take about 2-3 hours to complete.
2. Cognitive tasks involving attention, memory, and problem solving, as well as questionnaires about gambling activity and daily functioning that will take about 3 hours to complete.
3. A 10-week cognitive training program that can be easily accessed online.

**“HOW MANY PEOPLE WILL TAKE PART IN THIS STUDY?”**

81 people will take part in this study, which is only being conducted in Calgary.

**“WHAT WILL MY PARTICIPATION INVOLVE?”**

If you take part in this study, you will have the following tests and procedures:

- Interview and questionnaires asking about demographic information (e.g., age and education), and health history. This may include details of your past psychological and medical treatments along with any lifetime history of major medical disorders, as well as history of gambling.
- Measurements of current cognitive functioning.
- If you have already participated in a previous study in the CNS Laboratory, we ask that you consider allowing us to use your previous test results in the current study.
- You will be randomly assigned to either the 10-week cognitive training group or a 10-week wait-list control group. If you are assigned to the wait-list control group, your care will not be affected, and you will have the option of enrolling in the cognitive training program once the 10-week waiting period is over.

**“HOW LONG WILL I BE INVOLVED IN THE STUDY?”**

You will be involved in this study for a maximum of 3-5 hours per session, for a total of three sessions. Should you consent to being contacted by the CNS Laboratory in the future, you may be asked to participate in further studies, however these are considered separate studies, and your participation in them is completely voluntary.

**“WHAT ARE THE SIDE EFFECTS?”**

The risks involved in participation in this study are minimal. You may experience some fatigue or boredom in completing the cognitive tasks. You may feel uncomfortable when asked questions of a personal nature.

**UNIQUE SIDE EFFECTS /SPECIAL PRECAUTION**

N/A

“**WHAT ARE MY RESPONSIBILITIES?”**

You must be willing to attend scheduled study visits and participate in the study tasks. If you are assigned to the cognitive training group, you will also be asked to play the online brain training games for 30 minutes per day, 5 times per week, over a 10-week period.

**“WHAT ARE MY ALTERNATIVES?”**

You may choose not to participate in this study.

**“ARE THERE ANY BENEFITS TO PARTICIPATING IN THIS STUDY?”**

The current study is designed to test whether people with schizophrenia will benefit from an online ‘brain training’ program. Therefore, whether you will directly benefit from your participation in this study is to be determined. The purpose of this research is to gather scientific information. Knowledge produced from this work may help promote a better understanding of cognitive and daily functioning in people with schizophrenia.

**“CAN I WITHDRAW FROM THIS STUDY?”**

Taking part in this study is voluntary; you may withdraw from the study at any time if you wish to do so. Should you decide to withdraw from the study at any time, information collected from you until that point will still be used for the study unless you request your data be excluded from analyses.

**“WILL I BE PAID FOR PARTICIPATING, OR DO I HAVE TO PAY FOR ANYTHING?**

Compensation for your participation in this study will be $20 per session, which is intended to cover any additional costs to you for taking part in this study, such as transportation, meals, babysitting etc.

**“WHAT ARE MY RIGHTS AS A PARTICIPANT?”**

If you suffer an injury or become ill as a result of participating in this research, you will receive all medical treatments (or services) recommended by your doctors. No compensation will be provided beyond this point. However, it is important to note that nothing said in this consent form alters your legal rights to recover damages (e.g., legal action).

If new information becomes available or there are changes to the study that may affect your health or willingness to continue in the study, you will be told in a timely manner.

**“WILL MY PERSONAL INFORMATION BE KEPT CONFIDENTIAL?”**

All personally identifying information (e.g., name, date of birth etc.) will be removed from all assessment data collected. A master list connecting your data with your identity will be kept in confidence by the CNS Laboratory, and will be shared with only those researchers directly involved in the study.

The information collected during this study will be used in analyses and will be published and/or presented to the scientific community at meetings and in journals, but your identity will remain confidential. It is expected that the study results will be published as soon as possible after completion.

**“WHO DO I CALL IF I HAVE QUESTIONS OR PROBLEMS?”**

If you have any questions or want clarification regarding this research and/or your participation, please contact:

Dr. Vina Goghari

Department of Psychology, Clinical Psychology Program

(403) 210-7344 vina.m.goghari@ucalgary.ca

If you have any questions concerning your rights as a possible participant in this research, please contact The Chair of the Conjoint Health Research Ethics Board, University of Calgary, at 403-220-7990.

**UNDERSTANDING OF PARTICIPANTS**

I can refuse to take part or withdraw from this study at any time without jeopardizing my health care. If I continue to take part in the study, I will be kept informed of any important new developments and information learned after the time I gave my original consent.

I have read and understood all of the information in this consent form. I have asked questions, and received answers concerning areas I did not understand. I have had the opportunity to take this consent form home for review and discussion. My consent has not been forced or influenced in any way. I consent to participate in this research study. Upon signing this form I will receive a signed copy of the consent.

| I agree to take part in the cognitive training (randomized control) study | Yes | No |
| --- | --- | --- |
| If applicable, if I have recently participated in a study in the CNS Laboratory I agree to include my previous test data in the current study | Yes | No |
| I agree to be contacted to be told about future related research being conducted in the CNS Laboratory | Yes | No |

(PRINT NAMES CLEARLY)

_______________________ _____________________________ ___________________________

Name of Participant Signature of Participant Date

________________________ _____________________________ __________________________

Name of Person Signature of Person Date

Obtaining Consent Obtaining Consent
